# Healthcare Resource Utilization and Use of Biologics in Chronic Spontaneous Urticaria

**DOI:** 10.1111/1346-8138.70234

**Published:** 2026-04-04

**Authors:** Lillian D. Sun, Ajay Behl, Peter N. Danilov, Fatai Y. Agiri, Kathryn M. Pridgen, Amanda Christine F. Esquivel, Julie A. Lynch, Elma Baron

**Affiliations:** ^1^ Department of Dermatology Louis Stokes Cleveland VA Medical Center Cleveland Ohio USA; ^2^ B1 Health Corp New Jersey USA; ^3^ VA Informatics and Computing Infrastructure (VINCI), VA Salt Lake City Health Care System Salt Lake City Utah USA; ^4^ Division of Epidemiology, Department of Internal Medicine University of Utah School of Medicine Salt Lake City Utah USA

**Keywords:** biologics, chronic spontaneous urticaria, healthcare economics, healthcare utilization

## Abstract

Biologic therapies have emerged as effective treatments for chronic spontaneous urticaria (CSU), a debilitating skin disease. However, real‐world data on their use are limited. This retrospective descriptive study analyzes the time from diagnosis to biologic therapy initiation and the CSU‐specific healthcare resource utilization (HCRU) among patients with ≥ 2 urticaria diagnoses or one urticaria and one angioedema diagnosis with no diagnosis of urticarial vasculitis. This study uses data from the Veterans Health Administration from January 2011 through December 2021. We measured time from diagnosis to biologic initiation and CSU‐specific HCRU (outpatient visits, inpatient admissions, emergency room visits, and pharmacy claims) in the 12 month pre‐ and post‐index periods. The final cohort included 26 387 Veterans with CSU. In the 12 month post‐index period, 23 699 Veterans (89.8%) started treatment, but only 613 Veterans (2.6%) started biologic therapy, with a median initiation time of 337 days. CSU‐specific HCRU increased in the post‐index period across all categories. 66.8% of Veterans had pharmacy claims pre‐index date compared to 89.8% post‐index date, and 92.4% of Veterans had outpatient visits pre‐index date compared to 96.7% post‐index date. The findings suggest that initiation of biologics may be considered sooner in appropriate patients. The increased HCRU observed in the post‐index period highlights the burden that CSU places on patients and the healthcare system.

## 1 Introduction

Chronic spontaneous urticaria (CSU) is a debilitating skin disease characterized by recurrent wheals and/or angioedema for six weeks or longer without an identifiable external cause [1]. Clinical presentation includes persistent itching and pain, sometimes leading to disrupted sleep and chronic fatigue [2, 3]. This constellation of symptoms with unpredictable flare‐ups and a lack of a clear cause impacts patients daily, affecting work, social activities, and emotional well‐being. The psychological burden of CSU is profound, leading some to social withdrawal and a decreased quality of life [2, 4, 5].

Biologic therapies have emerged as an effective, guideline‐recommended treatment for moderate to severe CSU, particularly in patients unresponsive to conventional therapies [1, 6]. Real‐world data corroborates its effectiveness and safety [7]. However, real‐world data on treatment patterns, including biologic use, among patients with CSU are limited. This study addresses this gap by analyzing the time to biologic therapy initiation and healthcare resource utilization (HCRU) in Veterans with CSU receiving care through the Veterans Health Administration (VHA).

## 2 Methods

### 2.1 Data Source

This retrospective descriptive study leveraged data from the Corporate Data Warehouse (CDW), which provides access to longitudinal electronic health record data from the VHA, the largest integrated healthcare system in the United States.

### 2.2 Study Population

This study uses data from January 1, 2011, to December 31, 2021 and utilized an established algorithm to identify CSU cases [8]. Participants were ≥ 18 year with ≥ 2 urticaria diagnoses (ICD9 codes: 708.1, 708.8, 708.9; ICD10 codes: L50.1, L50.8, L50.9) or one urticaria and one angioedema (ICD9 code: 995.1; ICD10 code: T78.3) diagnosis. Diagnoses must have been made ≥ 6 weeks but ≤ 12 months apart. The Veterans were also required to have ≥ 12 months of continuous care before and after the index date, defined as the first date with a CSU diagnosis. Patients with urticarial vasculitis (ICD10 code: L95) or who were missing the date of birth or sex were excluded.

### 2.3 Data Analysis

We retrieved patient demographics at the index date and clinical characteristics of patients, including treatments received during the 12 month pre‐ and post‐index periods. The cohort was analyzed for time to biologic initiation and CSU‐specific HCRU (i.e., outpatient visits, inpatient admissions, emergency room visits, and pharmacy claims) in the 12 month pre‐ and post‐index periods. Data were quantified using descriptive statistics.

## 3 Results

### 3.1 Demographics

We identified 137 749 patients with a diagnosis of CSU during the study period. After applying inclusion and exclusion criteria, the cohort included 26 387 Veterans. We excluded those with a primary diagnosis of urticarial vasculitis at any time during the study period (*n* = 208) and those with a missing value for patient ID, year of birth, or sex (*n* = 2). The final cohort had a mean age of 54.9 years (SD = 15.2). Most patients were male (76.4%), though CSU was more common in women (0.37%) than men (0.18%). Among the cohort, 60.8% (*n* = 16 048) self‐identified as White and 28.0% (*n* = 7395) as Black (Table 1). Most Veterans with CSU were from the southern US (46.5%), followed by the west (19.9%), with 71.9% living in urban areas. Many were classified as overweight (33.3%, *n* = 8793) or obese (46.3%, *n* = 12 229), and 21.1% (*n* = 5566) were current smokers, highlighting lifestyle risk factors.

**TABLE 1:** Veteran Demographic Data.

Characteristics	Veterans with CSU (*N* = 26 387)
Age, mean (SD), years	54.9 (15.2)
Age category, *n* (%)	
18–29 years	1473 (5.6)
30–39 years	3913 (14.8)
40–49 years	4399 (16.7)
50–59 years	5557 (21.1)
60–69 years	6868 (26.0)
≥ 70 years	4177 (15.8)
Male, *n* (%)	20 149 (76.4)
Self‐reported race, *n* (%)	
White	16 048 (60.8)
Black	7395 (28.0)
Asian	723 (2.7)
Native Hawaiian/Pacific Islander	324 (1.2)
American Indian/Alaska Native	196 (0.7)
BMI, mean (SD), kg/m^2^	30.2 (6.3)
BMI category, *n* (%)	
Underweight: < 18.5 kg/m^2^	336 (1.3)
Normal weight: 18.5 to < 25 kg/m^2^	4731 (17.9)
Overweight: 25 to < 30 kg/m^2^	8793 (33.3)
Obese: ≥ 30 kg/m^2^	12 229 (46.3)
Smoking status, *n* (%)	
Current smoker	5566 (21.1)
Former smoker	6920 (26.2)
Never smoker	8961 (34.0)

### 3.2 Comorbidities

The most common comorbidities included hypertension (35.9%), sleep disorders (32.5%), depression (26.3%), anxiety (24.7%), diabetes (22.8%), inflammatory gastrointestinal disease (19.5%), allergic reactions (11.7%), asthma (11.0%), and chronic obstructive pulmonary disease (6.3%).

### 3.3 Treatment History

**FIGURE 1:**
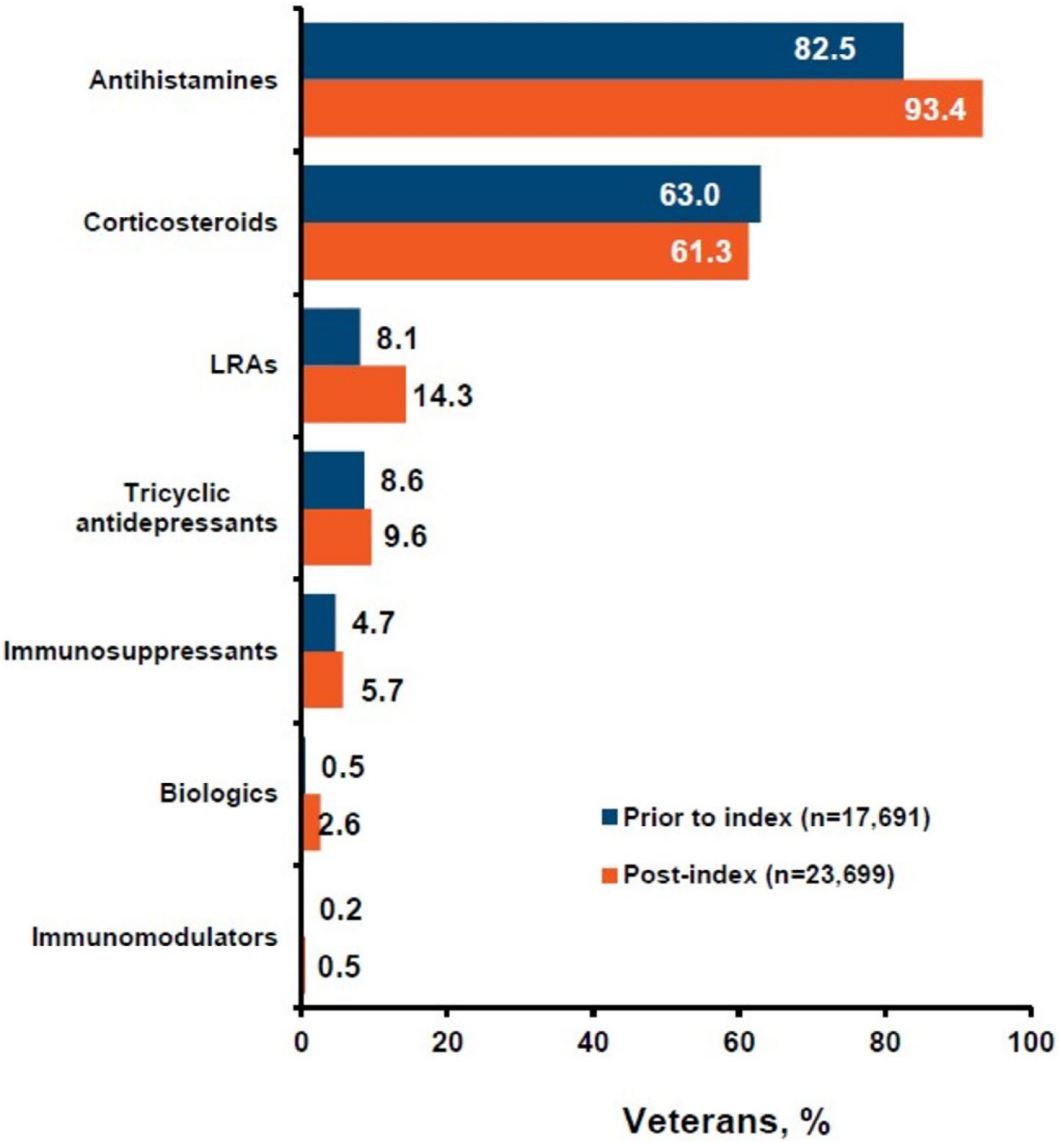
Pharmacological Treatments for Veterans with CSU.

In the 12‐month post‐index period, 23 699 Veterans (89.8%) started treatment. The most common treatments before and after the index date were antihistamines, corticosteroids, leukotriene receptor antagonists, tricyclic antidepressants, and immunosuppressants like cyclosporine (Figure 1). Only 613 Veterans (2.6%) started biologic therapy, with a median initiation time of 337 days.

### 3.4 Healthcare Resource Utilization

**FIGURE 2:**
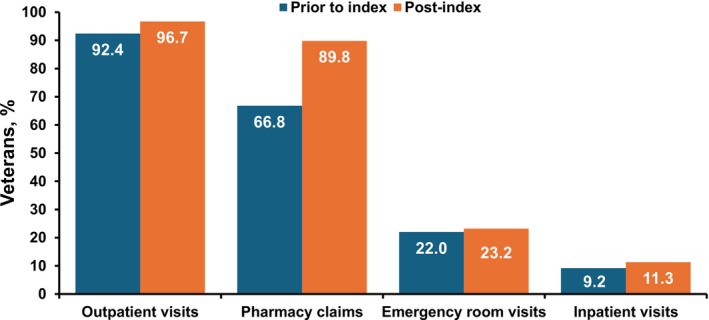
Healthcare Resource Utilization Among Veterans with CSU.

We measured HCRU by outpatient visits, inpatient admissions, ER visits, and pharmacy claims before and after CSU diagnosis. CSU‐specific HCRU increased notably in the post‐index period across all categories (Figure 2). 66.8% of Veterans had pharmacy claims pre‐index date compared to 89.8% post‐index date, indicating increased medication use. Outpatient visits were utilized by 92.4% of Veterans pre‐index date and 96.7% post‐index date, highlighting the need for ongoing care. Inpatient admissions and ER visits also rose, with the median length of stay increasing from four to five days, reflecting the potential severity of CSU episodes.

Further analysis of the number of post‐index CSU‐related ER visits (total = 4640) revealed a significant difference (*p* < 0.001) between men (3664 or 13.9% of all CSU cases; mean = 0.25, SD = 0.62) and women (976 or 3.7% of all CSU cases, mean = 0.20; SD = 0.57). Looking at age groups from 18‑29 up to 70 and above, patients between 50 and 69 years accounted for the highest number of CSU‐specific ER visits (51.9%). The differences between age groups were significant (*p* < 0.0001), as were the differences among races, with White patients showing the highest number of ER visits (56.3%) followed by Black patients (34.6%). All other races combined accounted for only 9.1%.

## 4 Discussion

These results highlight two key findings: the potential underutilization of biologic therapies and the significant healthcare resource burden associated with CSU management. The low percentage (2.6% within 12 months) of patients receiving biologics suggests continued reliance on other treatments, such as antihistamines and corticosteroids, despite the fact that antihistamines are insufficient for much of the population and corticosteroids likely increase adverse events [9, 10, 11]. While potentially useful in managing some symptoms of CSU, these drugs may fail to provide long‐term disease control for patients with moderate to severe CSU who need more targeted therapeutic strategies. Various factors may contribute to underutilization of biologics, including patient hesitancy towards injectable medications, limited awareness of newer treatment options, or provider's comfort level with prescribing biologics, which may greatly differ among primary care providers compared to specialists. Studies performed in different patient populations show a higher rate of biologic use (8%–33%) [12, 13, 14]. Unlike these other studies, however, our study only examined treatment patterns within the first year after diagnosis; more patients may have been prescribed biologics at a later date.

Similar to previous research [2], we found increased HCRU following diagnosis, underscoring the burden that CSU places on healthcare systems. The rise in pharmacy claims and outpatient visits indicates that some patients may experience persistent symptoms due to suboptimal disease control. Optimizing treatment through more targeted therapies could potentially result in better disease control, thus reducing the overall demand on healthcare resources. Significant differences in CSU‐specific ER utilization, peaking in the 50–69 year age group, suggest that closer follow‐up may be necessary among older patients. Notably, the most common comorbidities were chronic inflammatory diseases, which likely contributed to the ongoing need for medical care among these patients.

This study is limited by the retrospective nature of the analysis. Some Veterans may have sought care outside the VHA or moved between facilities. Additionally, the CDW sources VHA data from multiple platforms, potentially leading to incomplete information. We were unable to determine whether corticosteroids were prescribed short term or long term or whether HCRU was higher in patients with certain comorbidities. This Veteran patient population also differs from the general public in age, comorbidities, and socioeconomic factors, affecting generalizability. Finally, the Veteran population is primarily male, but CSU is more common among women [15].

## 5 Conclusion

This study offers valuable insights into real‐world treatment patterns and HCRU of patients with CSU. The findings suggest that management of CSU may be further optimized within the first year of diagnosis and initiation of biologics may be considered sooner in appropriate patients. Additionally, the increased HCRU observed in the post‐index period highlights the burden that CSU places on both patients and the healthcare system. Further research is needed to understand the barriers to biologic therapy initiation and to develop strategies to improve access to advanced treatment options for CSU. Optimizing treatment pathways for patients with CSU has the potential to improve patient outcomes and reduce the long‐term healthcare resource burden associated with this chronic condition.

## Funding

This work was supported by Novartis.

## Ethics Statement

This study received ethical approval from the Minneapolis and Salt Lake City VA Institutional Review Boards. All patient information was de‐identified and patient consent was not required.

## Conflicts of Interest

J.A.L., P.N.D., F.Y.A., and K.M.P. report grants from Alnylam Pharmaceuticals Inc., AstraZeneca Pharmaceuticals LP, Biodesix Inc., Janssen Pharmaceuticals Inc., Novartis International AG, and Parexel International Corporation through the University of Utah or Western Institute for Veteran Research outside the submitted work. A.B. was previously employed by Novartis. L.D.S., A.C.F.E., and E.B. have no disclosures to report.

## Data Availability

The data that support the findings of this study are available on request from the corresponding author. The data are not publicly available due to privacy or ethical restrictions.
